# Scleroderma renal crisis in tropical region: two senegalese cases

**DOI:** 10.11604/pamj.2015.21.46.6344

**Published:** 2015-05-22

**Authors:** Mouhamadou Moustapha Cisse, Sidy Mohamed Seck, Daher Abdoul Karim Oumar, Khodia Fall, Ahmed Tall Lemrabott, Moussa Diallo, Maria Faye, Moustapha Faye, Abdou Niang, Boucar Diouf

**Affiliations:** 1 Nephrology Department of Teaching Hospital, Aristide le Dantec, Dakar, Sénégal; 2 Nephrology Department of Gaston Berger University, Saint Louis, Sénégal; 3 Dermatology Department of Teaching Hospital Aristide le Dantec, Dakar, Sénégal

**Keywords:** Scleroderma renal crisis, corticosteroids, thrombotic microangiopathy, Dakar

## Abstract

Scleroderma renal crisis (SRC) is defined as the new onset of accelerated arterial hypertension and /or rapidly progressive oliguric renal failure during the course of systemic sclerosis. It is a rare but life-threatening complication. This formerly serious complication has got a considerable brighter outlook since the introduction of angiotensin converting enzyme inhibitors (ACE) however the mortality is still remaining high. We report two cases of SRC which to our knowledge are the firsts described in Dakar. They were two women aged 45 and 32 years, one of them was previously following for systemic sclerosis. Both of them had malignant hypertension associated with rapidly progressive renal failure, the other was put under corticosteroid therapy four months before SRC occurrence. The histological and laboratory finding showed thrombotic microangiopathy. The height blood pressure returned to normal value after treatment with ACE inhibitors. The final outcome was undesirable with the death of one after two months due to the hemodialysis discontinuation and persistence of renal failure in the other.

## Introduction

Systemic sclerosis (SSc) is a chronic multisystem autoimmune disease characterized by a vasculopathy. Scleroderma renal crisis (SRC) is defined as the new onset of accelerated arterial hypertension and /or rapidly progressive oliguric renal failure during the course of systemic sclerosis [1]. It occurs in approximately 1.6 to 5.3% of SSc [2]. The mortality of SRC is high so that renal involvement remains a constant concern during the following of scleroderma patients. In Africa renal involvement is exceptionally reported and few cases of SRC have been described. We report two cases of SRC that to our knowledge are the first two descriptions in Dakar.

## Patient and observation

### Case N°1

**Figure 1:**
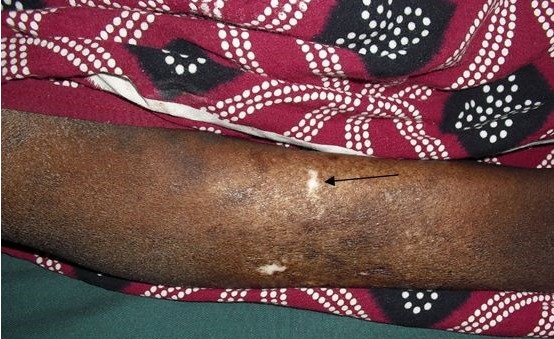
Salt and paper hypopigmentation (arrow)

**Figure 2:**
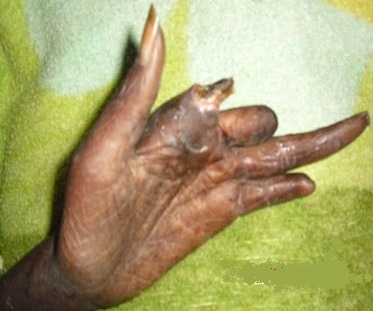
Left hand finger cut off

**Figure 3:**
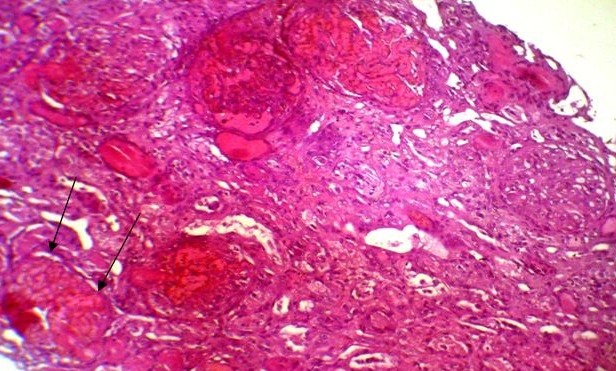
Partial cortical necrosis (arrows: destructed glomerulus). HES (x100)

**Figure 4:**
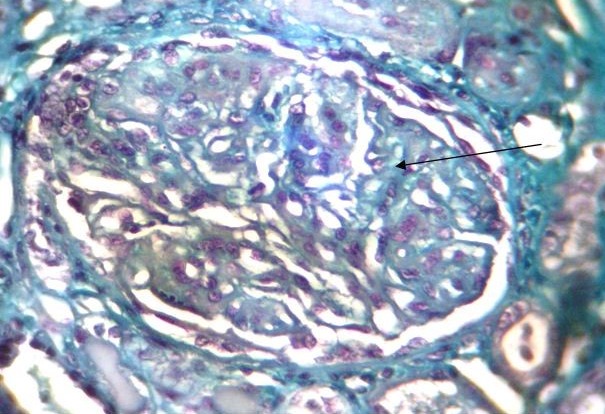
Hemorrhagic mesangiolysis (arrow) glomerular thrombotic microangiopathy. Trichrome de masson (x100)

**Figure 5:**
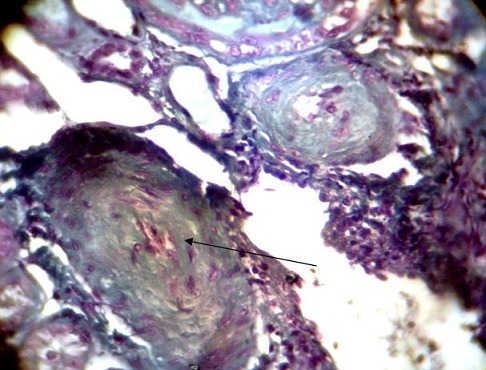
Arteriolar lesions in “onion skin” (arrow). Trichrome de masson (x100)

A 45 year's old Senegalese woman with systemic sclerosis was referred to internal medicine department for a height blood pressure of 230/140 mm Hg, oliguria of 150 cc/day and dyspnea. Past medical history revealed that she was following in dermatology department since 1996 when she was presenting clinical and laboratory signs and symptom of systemic scleroderma such as Raynaud's phenomenon, non-deforming polyarthritis of small joints, gastroesophageal reflux. sclerodactyly, salt and pepper hypopigmentation (Figure 1), multiple amputated fingers (Figure 2), face skin sclerosis a sharp nose, positive Anti Scl 70 antibodies and a normochromic normocytic anemia with Hb of 8 g / dl. RNA polymerase III antiantibodies had not been requested. The patient had been clinically stable with mild hypertension of 13.5/8.5 and a normal renal function was noted until 2008 when she was lost to follow. In 2011 the patient consulted again in dermatology department for weight loss and poor health status. The patient was put under treatment with diltiazem 60 mg / day and prednisone 40 mg / day. Four months later the patient was referred to nephrology department for height blood pressure and oliguria. On admission, BP was 230/140 mm Hg and 24 hours urine collection revealed an oliguria with 150 cc / day. Cardiovascular examination showed congestive heart failure and retinal examination disclose a stage III of hypertensive retinopathy. Dipstick urine analysis showed proteinuria of 3+ and a 2 + hematuria. 24 hours proteinuria was 3.5 g. CBC revealed a normochromic normocytic anemia with an Hb of 7g/dl, a thrombocytopenia of 99 000 /mm^3^ and 2.5% schisocytes in blood smear. Renal function test was impaired with a creatinine of 156 mg /L and blood urea of 1.76 g/L. a hyperkalemia of 6.7 mEq /L and sodium of 139 mEq /L also detected. Lactate dehydrogenase (LDH) was 3 times elevated than normal. Renal sonography showed normal size kidneys with a poor corticomedullary differentiation. The patient was quickly gone under treatment with Nicardipine IV perfusion and hemodialysis. Later the patient was transfused and captopril 25 mg TID instituted. Prednisone started to be tapered and discontinued. After controlling of hypertension and thrombocytopenia correction, a renal needle biopsy was performed. Histopathologic examination of biopsy yield specimen showed a partial cortical hemorrhagic necrosis (Figure 3) associated with edematous thickening of mesangium extending to the glomerular basement membrane (see Figure 4). The tubulointerstitial involvement was dominated by foci of tubular epithelial necrosis (Figure 3) and vascular involvement marked by the presence of arteriolar thrombosis, fibrointimal proliferation and “onion skin” lesions (Figure 5).

### Case N°2

A 32 years old woman without any remarkable past medical history was hospitalized for dyspnea stage IV, low consciousness level without fever and focal neurological signs and sudden onset oliguria, five days after a normal vaginal delivery. The review of systems highlighted a diffuse arthralgia, a Raynaud's phenomenon and a thickening of fingers skin since one a year ago. Physical examination on admission showed a BP of 250/130 mm Hg, a heart rate of 110 bpm, a respiratory rate of 30 cycles /min, temperature 36.5°C, weight 45 kg, beaklike facies, sausage shape fingers and toes associated with salt and pepper hypopigmentation localized on the abdomen and back. Laboratory data revealed a severe renal insufficiency with a creatinine of 48.5mg/l, normocytic anemia to 8.1g/dl associated with thrombocytopenia 80,000 / mm^3^ and positive anti-nuclear and anti-scl 70 antibodies.

Ultrasonography revealed a poor corticomedullary differentiation, echogenic renal cortices without pelvicalyceal system distention. Chest x ray displayed a cardiomegaly and bilateral reticulonodulaire lesions predominantly in the lower segments of the right lung. Echocardiography showed severe pulmonary hypertension and left ventricular dysfunction. No central or peripheral vascular calcification was noticed. The patient underwent treatment with captopril and hemodialysis in the same time. The blood pressure decreased but renal functions did not improve. Renal biopsy disclosed a thrombotic microangiopathy. We could not put the patient under immunosuppressive therapy because of patient's low income. After three months of therapy with dialysis, the patient had an ischemic necrosis of the right second toe. Finally the patient was withdrawn from dialysis after a partial recovery of renal function.

## Discussion

The SRC is known since 1863, but its histopathologic aspects were first described in 1952 [3]. In 1970 estimated prevalence was between 12 to 18% [4]. The age of onset varies between 43 and 64 [5–8], these data fit with those of the patient No. 1 who was 45 years although the case No 2 of 32 years old was younger. There are Risk factors for SRC (Table I) [2, 5, 9]. Indeed, in a study by Stenn et al 25% of the patients had diffuse cutaneous involvement [2]. Both of our patients had cutaneous sclerosis with a slow evolution over several years for the case NO 1 and this is partly explained by the age of onset (16 years) of the SRC. Anemia especially a newly developed is also considered a risk factor as it was the case for our two patients with a hemoglobin of 8 and 8.1 g / dl. Zbiti also reported a normocytic normochromic anemia with hemoglobin of 6g/dL [5]. High-dose corticosteroid therapy longer than 3 months is considered as a powerful risk factor of SRC [6, 8–11] as evidenced by case No 1 who developed a SRC after 4 months treatment with 40 mg of prednisone per day. Hypertension, usually malignant (DBP ≥ 130 mm Hg) was found in approximately 87% of patients [6, 8] in its typical form. In our study 2 patients had malignant hypertension one a BP of 230 / 140mmHg and hypertensive retinopathy (case NO 1) and the other with a BP of 250 / 130mmHg and low consciousness level (case NO 2).

The histologic picture of SRC is a thrombotic microangiopathy which is seen in 43 to 56% [6, 8]. A process which mainly involves small vessels [12] and manifests as myxoid intimal changes, thrombi, onion skin lesions, and/or fibrointimal proliferation. Renal biopsies, even though necessary to confirm the diagnosis, are not routinely warranted in SRC. It must be done very carefully when the blood pressure is fully controlled and the platelet count is normal otherwise transjugular renal biopsy is more appropriate [5, 13]. Prevention is the first step of SRC treatment. Although it is not clinically proven but the authors propose the use of angiotensin converting enzyme inhibitors (ACE) as a preventive measure in patients with diffuse skin [6, 8, 9]. The mainstay of therapy is to control blood pressure as soon as possible in the first 3 days with a goal of ≤ 120/80 mm Hg. [9] Angiotensin II receptor antagonist may be less effective [6]. Half of the cases will require hemodialysis [14, 15]. Both of our patients as well as 53.8% patients in Guillevin's study were put on hemodialysis [9].

Treatment should be promptly started, especially in case of poorly controlled hypertension or rapid deterioration of renal function. [10] Kidney transplantation is generally considered after a period of two years as at least a partial recovery of renal function is possible up to 2 years on dialysis [16, 17]. Preventive use of ACE is proposed after renal transplantation [18, 19]. Despite adequate care, the survival of patients with SRC remains low with an overall rate of 1, 2, 5 and 10 years survival for 70.9%, 66.6%, 60% and 41.9% respectively [8]. In our cases, the patient No 1 died following hemodialysis discontinuation because of financial reasons. A partial recovery of renal function and dialysis withdrawal was noted in case No. 2.

## Conclusion

These observations show the severity of scleroderma renal crisis. All risk factor should be avoided and regular measurement of blood pressure and serum creatinine should be a part of routine cares of patients with systemic sclerosis. A prospective large-scale study including all scleroderma patients should be considered in order to assess the prevalence and severity of scleroderma renal crisis in genetically pigmented people living in the tropical regions.
